# Supplementary material for: The Healthcare Experience of Autistic Patients in Orthopaedic Surgery and Closely Related Fields: A Scoping Review
Source: Children (Basel). 2023 May 22;10(5):906. doi: 10.3390/children10050906 (PMC10217637; doi:10.3390/children10050906)
Supplement: Supplementary file 1 [file children-10-00906-s001.zip › children-2403341-supplementary.pdf]

Supplement S1. Literature search query formula.

Last updated: March 3, 2023

Total: 122

Total to screen after deduplication: 114

Concept 1: Patients with Autism Spectrum Disorder

PubMed MeSH

"Autism Spectrum Disorder"[Mesh] OR ("Child Development Disorders, Pervasive"[Mesh:NoExp] AND 2010[pdat]:2015[pdat])

CINAHL

(MH "Autistic Disorder") OR (MH "Asperger Syndrome")

Embase

'autism'/exp

Keywords:

autistic OR autism OR asperger OR asperger's

Concept 2: Patient Experience

PubMed MeSH

("Treatment Adherence and Compliance"[Mesh]) AND ("Patient Compliance"[Mesh:NoExp] AND 1971[pdat]:2017[pdat])

CINAHL

(MH "Patient Compliance") OR (MH "Patient Satisfaction")

Embase

'patient attitude'/exp

Keywords:

patient experience* OR patient satisfaction OR patient compliance OR patient adherence OR patient preference*

(therapy OR treatment OR patient) NEAR/2 (satisfaction OR experience OR compliance OR adherence OR preference)

Concept 3: Movement Science

PubMed MeSH

((((("Physical and Rehabilitation Medicine"[Mesh]) OR ("Physical Therapy Specialty"[Mesh] OR "Physical Therapy Modalities"[Mesh])) OR "Occupational Therapists"[Mesh]) OR "Orthopedic Surgeons"[Mesh]) OR "Orthopedics"[Mesh]) OR "Orthopedic Procedures"[Mesh] OR "Gait"[Mesh]

CINAHL

(MH "Orthopedic Surgery") OR (MH "Orthopedics") OR (MH "Occupational Therapy") OR (MH "Physical Therapy") OR (MH "Gait")

Embase

'orthopedic surgery'/exp OR 'orthopedics'/exp OR 'physiotherapy'/exp OR 'occupational therapy'/exp OR 'rehabilitation medicine'/exp OR 'gait'/exp

Keywords:

"toe walking" OR "postural instability" OR gait OR "walking pattern" OR posture OR motor OR orthopedic* OR "occupational therap*" OR "physical therap*" OR "physical rehab*" OR

"rehabilitation medicine" OR "rehabilitation science" OR physiatry OR orthopaedic* OR physiotherapy OR ergotherap* OR "physical medicine"

'toe walking' OR 'postural instability' OR gait OR 'walking pattern' OR posture OR motor OR orthopedic* OR 'occupational therap*' OR 'physical therap*' OR 'physical rehab*' OR 'rehabilitation medicine' OR 'rehabilitation science' OR physiatry OR orthopaedic* OR physiotherapy OR ergotherap* OR 'physical medicine'

motor NEAR/2 (development OR delay OR difference OR abnormal*)

Queries:

PubMed

1 ("Autism Spectrum Disorder"[Mesh] OR ("Child Development Disorders, Pervasive"[Mesh:NoExp] AND 2010[pdat]:2015[pdat])) OR (autistic[Title/Abstract] OR autism[Title/Abstract] OR asperger[Title/Abstract] OR asperger's[Title/Abstract])

2 (("Treatment Adherence and Compliance"[Mesh]) AND ("Patient Compliance"[Mesh:NoExp] AND 1971[pdat]:2017[pdat])) OR (patient experience*[Title/Abstract] OR patient satisfaction[Title/Abstract] OR patient compliance[Title/Abstract] OR patient adherence[Title/Abstract] OR patient preference*[Title/Abstract])

3 (((((("Physical and Rehabilitation Medicine"[Mesh]) OR ("Physical Therapy Specialty"[Mesh] OR "Physical Therapy Modalities"[Mesh])) OR "Occupational Therapists"[Mesh]) OR "Orthopedic Surgeons"[Mesh]) OR "Orthopedics"[Mesh]) OR "Orthopedic Procedures"[Mesh] OR "Gait"[Mesh]) OR ("toe walking"[Title/Abstract] OR "postural instability"[Title/Abstract] OR gait[Title/Abstract] OR walking pattern[Title/Abstract] OR posture[Title/Abstract] OR motor[Title/Abstract] OR orthopedic*[Title/Abstract] OR occupational therap*[Title/Abstract] OR physical therap*[Title/Abstract] OR physical

rehab*[Title/Abstract] OR rehabilitation medicine[Title/Abstract] OR rehabilitation science[Title/Abstract] OR physiatry[Title/Abstract] OR orthopaedic*[Title/Abstract] OR physiotherapy[Title/Abstract] OR ergotherap*[Title/Abstract] OR physical medicine[Title/Abstract])

4 1 AND 2 AND 3

5 results

Embase

1 'autism'/exp

2 autistic:ti,ab,kw OR autism:ti,ab,kw OR asperger:ti,ab,kw OR aspergers:ti,ab,kw

3 1 OR 2

4 'patient attitude'/exp

5 ((therapy OR treatment OR patient) NEAR/2 (satisfaction OR experience OR compliance OR adherence OR preference)):ti,ab,kw

6 4 OR 5

7 'orthopedic surgery'/exp OR 'orthopedics'/exp OR 'physiotherapy'/exp OR 'occupational therapy'/exp OR 'rehabilitation medicine'/exp OR 'gait'/exp

8 (motor NEAR/2 (development OR delay OR difference OR abnormal*)):ti,ab,kw

9 'toe walking':ti,ab,kw OR 'postural instability':ti,ab,kw OR gait:ti,ab,kw OR 'walking pattern':ti,ab,kw OR posture:ti,ab,kw OR motor:ti,ab,kw OR orthopedic*:ti,ab,kw OR 'occupational therap*':ti,ab,kw OR 'physical therap*':ti,ab,kw OR 'physical rehab*':ti,ab,kw OR 'rehabilitation medicine':ti,ab,kw OR 'rehabilitation science':ti,ab,kw OR physiatry:ti,ab,kw OR

orthopaedic*:ti,ab,kw OR physiotherapy:ti,ab,kw OR ergotherap*:ti,ab,kw OR 'physical medicine':ti,ab,kw

10 7 OR 8 OR 9

11 10 AND [embase]/lim

CINAHL

1 (MH "Autistic Disorder") OR (MH "Asperger Syndrome")

2 TI (autistic OR autism OR asperger OR asperger's) OR AB (autistic OR autism OR asperger OR asperger's)

3 1 OR 2

4 (MH "Patient Compliance") OR (MH "Patient Satisfaction")

5 TI ((therapy OR treatment OR patient) N2 (satisfaction OR experience* OR compliance OR adherence OR preference*)) OR AB ((therapy OR treatment OR patient) N2 (satisfaction OR experience* OR compliance OR adherence OR preference*))

6 4 OR 5

7 (MH "Orthopedic Surgery") OR (MH "Orthopedics") OR (MH "Occupational Therapy") OR (MH "Physical Therapy") OR (MH "Gait")

8 TI (motor N2 (development* OR delay& OR difference* OR abnormal*)) OR AB (motor N2 (development* OR delay& OR difference* OR abnormal*))

9 TI ("toe walking" OR "postural instability" OR gait OR "walking pattern" OR posture OR motor OR orthopedic* OR "occupational therap*" OR "physical therap*" OR "physical rehab*" OR "rehabilitation medicine" OR "rehabilitation science" OR physiatry OR orthopaedic* OR physiotherapy OR ergotherap* OR "physical medicine") OR AB ("toe walking" OR "postural

instability" OR gait OR "walking pattern" OR posture OR motor OR orthopedic* OR "occupational therap*" OR "physical therap*" OR "physical rehab*" OR "rehabilitation medicine" OR "rehabilitation science" OR physiatry OR orthopaedic* OR physiotherapy OR ergotherap* OR "physical medicine")

10 7 OR 8 OR 9

11 3 AND 6 AND 10
